# Retraction: *miR-135a* promotes gastric cancer progression and resistance to oxaliplatin

**DOI:** 10.18632/oncotarget.28835

**Published:** 2026-03-31

**Authors:** Lin-Hai Yan, Zhi-Ning Chen, Li-Li, Jia Chen, Wen-E Wei, Xian-Wei Mo, Yu-Zhou Qin, Yuan Lin, Jian-Si Chen

**Affiliations:** ^1^Department of Gastrointestinal Surgery, Affiliated Tumor Hospital of Guangxi Medical University, Nanning 530021, Guangxi Zhuang Autonomous Region, China; ^2^Department of Pathology, Affiliated Tumor Hospital of Guangxi Medical University, Nanning 530021, Guangxi Zhuang Autonomous Region, China; ^3^Department of Pharmacy, The People Hospital of Guangxi Zhuang Autonomous Region, Nanning 530021, Guangxi Zhuang Autonomous Region, China; ^4^Department of Medical Image Center, Affiliated Tumor Hospital of Guangxi Medical University, Nanning 530021, Guangxi Zhuang Autonomous Region, China; ^5^Department of Research, Affiliated Tumor Hospital of Guangxi Medical University, Nanning 530021, Guangxi Zhuang Autonomous Region, China

**This article has been retracted:** Oncotarget has completed its investigation, and image forensic analysis confirmed extensive internal and external duplications across both main and supplemental figures. Internal duplications were identified in Figures 1A, 2A, 3A and 3E, 4D, 5C, 7E, 8B and 8D, and 9A, as well as in Supplementary Figures 1A and 3. External duplications were also found: Figure 4D duplicates Figure 2C in reference [1], Figure 8D duplicates Figure 6F in reference [2], Figure 6A–6C duplicates Figure 5B, 5D, 5F and Figure 5B duplicates Figure 4C in reference [3], respectively. Accordingly, the Editorial Decision was made to retract the publication. While one of the corresponding authors, Dr. Lin-Hai Yan, requested the retraction, we did not receive the formal retraction agreement with the signatures of all authors.

Original article: Oncotarget. 2016; 7:70699–70714. 70699-70714. https://doi.org/10.18632/oncotarget.12208
